# Long-Term Survival of Dental Implants Placed in the Grafted Maxillary Sinus: Systematic Review and Meta-Analysis of Treatment Modalities

**DOI:** 10.1371/journal.pone.0075357

**Published:** 2013-09-18

**Authors:** Fabian Duttenhoefer, Cyriel Souren, Dieter Menne, Dominik Emmerich, Ralf Schön, Sebastian Sauerbier

**Affiliations:** 1 Department of Oral and Craniomaxillofacial Surgery, University Hospital Freiburg, Freiburg, Germany; 2 Menne Biomed Consulting, Tübingen, Germany; 3 Department of Oral and Craniomaxillofacial Surgery, Malteser Krankenhaus St. Josefshospital, Krefeld-Uerdingen, Germany; Department of Biomaterials, Japan

## Abstract

**Background:**

A prevalent modality to increase the amount of available bone prior to implantation is grafting of the maxillary sinus. Multiple factors such as the surgical technique, moment of implant placement as well as grafting materials and membranes are known to affect implant survival. However, the role of different factor combinations and associated reciprocal effects remain unclear. Conventional statistical methods do not consider inconsistency of study designs and do not take covariables into account. Hence, a systematic research and meta-analysis was conducted to investigate the influence of various treatment modalities on implant survival in the grafted maxillary sinus.

**Materials and Methods:**

A meta-analysis was conducted according to the PRISMA guidelines. Articles published from 1980 through January 2013 were electronically and manually searched in MEDLINE (Ovid), the Cochrane Register of Controlled Trials, the Database of Abstracts of Effects, and the Cochrane Database of Systematic Reviews. Clinical reports on single intervention sinus augmentation with root-form implants, a minimum of 10 patients and 6 months of loading were eligible for inclusion if implant survival was stated or calculable. Results were calculated by non-parametric univariate Kaplan-Meier analysis and Bayesian multivariate interval-censored Cox regression.

**Results:**

A total of 122 publications on 16268 endosseous implants placed in grafted maxillary sinus were included. The treatment parameters surgical approach, grafting material and implant type showed no selective preference. However, application of membranes showed a significantly reduced hazard-ratio, independent of other co-factors.

**Conclusions:**

The use of membranes is the most significant factor to achieve long-term implant survival in sinus augmentation procedures. More data exceeding 3 years follow-up are needed to address prospective confounding and improve clinical evidence.

## Introduction

In a continuously growing and aging society the need for implant based dento-maxillary rehabilitation is expanding. Besides aesthetic recovery, the regeneration of the physiological function of the dento-maxillary system is crucial for adequate nutrition and improvement in quality of live. Concurrently, dento-maxillary rehabilitation has a considerable effect on the overall morbidity especially in elderly people, and a resultant socio-economic impact [1,2]. Vogel et al. 2013 could directly link successful dental implantology in senior citizens with improved overall health, quality of life and decreased health care costs [3]. Still, the predominant challenge for successful dento-maxillary rehabilitation is residual bone, the pivotal factor in patients subjected to maxillary implantation. Advanced atrophy of the alveolar crest, primarily in patients with edentulous or partially edentulous posterior maxillae impairs conventional insertion of implants and restoration of the dento-maxillary system. Accordingly health-care decision-making vastly depends on valid clinical evidence to assess the most beneficial treatment modalities.

To date, based on the conventional sinus floor elevation (CSFE) developed over three decades ago [4] numerous successful techniques have been described to restore maxillary bone height [5,6]. Most techniques feature a lateral approach to the sinus cavity. A horizontal incision is made in the mucosa at the top of the alveolar crest or slightly palatally to lift a full-thickness flap that is deflected to expose the lateral antral wall of the maxillary sinus. A bony window is produced utilizing either a round bur or piezotome to expose the Schneiderian membrane. The membrane is carefully detached from the walls of the maxillary antrum creating a void for augmentation. The grafting material is firmly inserted in the cavity and subsequently the deflected mucoperiosteum flap closes the sinus window. Several approaches involve classification and treatment of membrane tearing as well as adaptations to the closure of the sinus and the time point of implantation [7-10]. Today the CSFE presents a clinically successful technique that offers good insight into the sinus cavity and the present changes in bone height [11]. However, these advantages involve a secondary surgery site when placing dental implants and thus hold several drawbacks such as the potential for infections [12], particularly in smokers [13]. To address these drawbacks Summers et al. 1994 described a crestal approach to elevate the Schneiderian membrane utilizing tapered osteotomes with increasing diameters [14]. The basic procedure involves a crestal incision at the planned implant site and a full-thickness flap that is prepared to expose the alveolar crest. The initial osteotomy is either created manually with osteotomes or by the use of a bur or a drill. The subsequent osteotomes are inserted into the initial osteotomy by hand pressure or gentle malleting until the residual bone height beneath the maxillary sinus floor is limited to about 2 mm. Simultaneously, the diameter of the osteomes is consecutively increased until the planned implant diameter is reached. The last used osteotome is reinserted and pushed upwards to fracture the sinus floor and raise the Schneiderian membrane. To minimize the risk of membrane perforation some clinicians use an inflatable device or fill the void with augmentation material prior fracturing the sinus wall [15].

Today, several modifications of the operational technique have been described [16,17] but in most cases implant insertion is performed simultaneously after the desired augmentation height is reached. Most authors make their decision whether to use a simultaneous or staged approach according to the amount of residual bone height [18-24]. The consensus for simultaneous implant placement in grafted bone is limited to a residual bone height of at least 4-5 mm. Contrary, recent studies indicated successful one-stage approaches with only 1 mm residual bone height [25,26]. Taken together, the osteotome technique may provide lower morbidity and operational time but requires greater residual bone height. Furthermore, the osteotome technique is associated with a higher possibility of membrane tearing, limited elevation of the sinus mucosa and fewer control of the operation field [11]. Apart from the different surgical approaches providing adequate structure for primary implant stability several additional parameters such as simultaneously or delayed implant placement, time of unloaded healing as well as the use of grafting materials or membranes significantly affect implant survival. The ideal graft material is described as a substance that will change into regular bone under functional loading without resorption and offers the ability to form new bone either osteoconductively or osteoinductively to enable the support of dental implants [24,27].

A broad variety of different grafting materials has been successfully applied in sinus augmentation, including autogenous bone, allografts, xenografts and alloplasts. Autogenous bone is highly osteogenic, provides osteoprogenitor cells, disposes osteoinductive factors [23,24,28-30], and can be harvested from various donor sites (i.e. ilium, symphysis, mandibular ramus). Still, it was shown that autogenous bone is prone to high resorption [27,31], with up to 49.5% of bone loss after six months [32]. Additionally, the use of autogenous bone usually involves a second surgery site with the potential of donor site morbidity [24,27,28,30,32-34]. Allografts such as demineralized freeze-dried bone (DFDBA) avoid a second surgical site and exhibit osteoinductive and osteoconductive properties [28,35,36]. However, it was stated that DFDBA generates unpredictable bone formation with newly-formed bone of low quality and quantity [27,34,37]. The use of xenografts such as bovine bone mineral [38] and alloplasts such as hydroxyapatite [39] alone or in combination with autogenous bone, has increased over the past decade. Suchlike bone substitute materials vary in porosity and structure (particular pieces or blocks). Supplementary, some clinicians apply resorbable or non-resorbable membranes to shield the augmented area and prevent soft tissue encleftation. Thus, membranes may provide guided bone regeneration (GBR) and increase the amount of newly-formed bone [22,40,41]. On the other hand membranes may result in lower vascular supply to the graft, increased risk of infection [42], and additional cost. Furthermore, non-resorbable membranes need to be removed in a second surgery [40]. Peleg stated in 1999, that particulated grafts, which contain autogenous bone, heal faster and implants can be placed earlier [43]. Other authors [33,44] reported about a more favourable result for the use of xenografts. More recent investigations found similar survival rates for autogenous bone and bone substitutes [23,31] or stated that autogenous bone is still the gold standard [45]. Although sinus augmentation has become a frequently used and clinically successful technique the review of clinical investigations on sinus augmentation is inconsistent and often confounding [46]. Aghaloo and Moy 2007 stated that variations in the selection of patients, the surgical procedures as well as the surgeons skill-level account for the low clinical evidence. Consequently, the aim of this meta-analysis was to detect the predictability of sinus augmentation on the basis of implant survival and to compare the impact of the various treatment modalities. Particular attention was given to the influence of the surgical approach, residual bone height, the type of implant, its surface and placement, the grafting material and the use of membranes to provide clinical evidence for prospective treatment regimes.

## Materials and Methods

A MEDLINE (Ovid) search was conducted for articles published from 1980 up to January 2013. The Cochrane Register of Controlled Trials, the Database of Abstracts of Effects, and the Cochrane Database of Systematic Reviews were also electronically searched. The search terms ‘sinus’, ‘sinus floor’, ‘maxillary sinus’, ‘maxillary sinus floor’, ‘maxillary antrum’, AND ‘elevation’, ‘augmentation’, ‘graft’, ‘lift’, ‘bone transplant’, ‘bone graft’, ‘bone remodel’, ‘alveolar ridge augmentation’, AND ‘dental implant, ‘endosseous implant’, were combined with the text words ‘sinus floor elevation’, ‘sinus floor augment’, ‘sinus floor graft’, ‘sinus floor lift’, ‘sinus elevation’, ‘sinus augment’, ‘sinus graft’, and ‘sinus lift’. Limits were set to human trials. The meta-analysis was conducted according to PRISMA guidelines and the methods of the analysis and inclusion criteria were specified in advance by the reviewers and documented in a protocol (Checklist S1). The initial criteria were not subjected to any alterations throughout the study:

1 Clinical report
2 No abstract publications
3 Studies in English and German
4 Absence of multiple interventions
5 Root-form implants
6 Minimum of 6 months follow up after implant loading
7 Minimum of 10 patients
8 Implant success/survival either clearly reported or calculable

Study selection and data extraction was performed by three independent reviewers. Uncertainties were discussed among the authors.

Information was extracted from each included trial on: (1) publication year, (2) study type (including case series, case control studies, cohort studies and randomized controlled trials), (3) implant form (screw versus pressfit), (4) implant surface (rough versus machined), (5) graft material (including autogenous bone; or bone substitute; or a combination of these two; or no graft at all), (6) the use of a membrane or not, (7) placement of the implants (one-stage versus two-stage), (8) residual bone height (9), unloaded healing time of the implants (10), surgical technique (lateral window versus transcrestal approach), and (11) type of outcome measure (survival versus success criteria). The authors of 78 studies were contacted by mail to clarify missing, insufficient, inadequate or controversial data. Studies with continuous unclear or incomplete data were excluded.

### Statistical analysis

Numerical analysis was done, and plots and tables were generated with R. [47] The number of surviving implants at fixed intervals up to 8 years post-implantation had been entered in an Excel (Microsoft) spread sheet. Since the exact event times were not known, the events were considered interval-censored for lost implants, and right-censored for surviving implants. For interval-censored data, it is assumed that the probability of dropout in the interval is a smooth function over the time spanned by two consecutive visits. To follow intention-to-treat principles, implant loss during surgery was coded as uncensored loss on day 1 after implant. Non-parametric univariate Kaplan-Meier analysis was done with the R package *interval* [48]. Multivariate analysis used a Bayesian approach as implemented in package *dynsurv* [49] to separate the effect of publication from that of the implantation methods. The results are given as log-hazard ratios (HR) relative to a median baseline hazard. Forest plots summarizing the results of meta-analysis were created with package *meta* [50]. Because survival data were available for only few of the possible combinations of the parameters, strong priors were used to stabilize Bayesian posteriors. For the log-hazard coefficients, the prior had a mean of 0 and a standard deviation of 0.3; for the lambda coefficients defining the baseline hazard, a gamma distribution with shape = 6 and rate = 10 was chosen. Base levels and all pairwise interactions of parameters *Membrane*, *Approach* and *Implant* were used as predictors in the multivariate Bayes model; for the 4 levels of the graft material (*GraftMat*), no interactions were included because too few combinations were known. In addition for each publication a relative log hazard was estimated, to correct the estimation of the method hazard for study specific effects. In terms of the R-programming language, the following model formula was used:

Survival ~ (Implant+Membrane+Approach)^2+GraftMat+Publication

Means and 95%-confidence intervals of log-hazards were computed from 30000 MCMC samples after 3000 burn-in samples; a typical model run required 5 hours of computing time [50]. In practice, the natural logarithm of the hazard ratio is a more useful measure and was used throughout this paper. Positive log hazard ratios stand for steeper survival curves and shorter survival time.

## Results

The electronic search provided a total of 1960 duplicate adjusted citations. Of these, 1682 were discarded because after reviewing the abstracts it appeared that these papers clearly did not meet the inclusion criteria. The full text of the remaining 278 citations was examined in more detail. It appeared that 156 studies did not meet the inclusion criteria as described. 122 studies met the inclusion criteria and were included in the systematic review.

**Figure 1:**
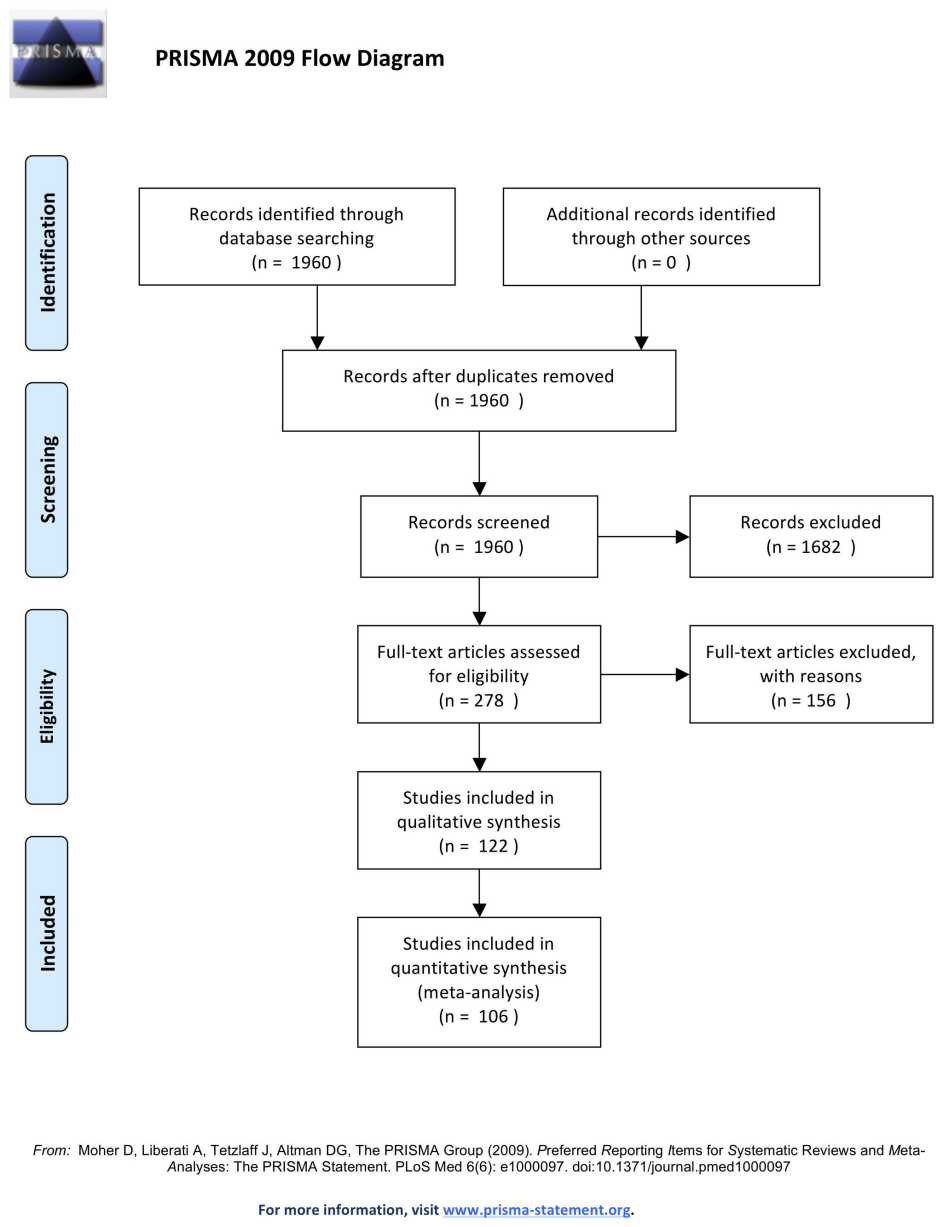
PRISMA – Flow Diagram.

A total of 122 publications on 16268 endosseous implants placed in grafted maxillary sinus covering publication years 1993 to 2012 were shortlisted for meta-analysis (Figure 1). The implant parameter descriptors were extracted from the publications (Table 1). In case a publication reported data from multiple methods, results were subdivided into *References* disambiguated by appending *_a*, *_b* or *_c* (table 2) and further itemized (Table 3). The included publications showed a marked annually increase in number of studies and implants after the year 2004. Follow-up time for most publications was 1 year, with only one study investigating 11 years follow-up time [51]. Information on parameters *Approach*, *StudyType, Placement* and *GraftMat* is available for all publications. In a preliminary one-factorial analysis, parameters *StudyType* and *Placement* showed to have no predictive value and were therefore omitted. Since only 4% of the applied implant surfaces were machined, parameter *Surface* was also omitted to avoid power reduction. Parameter *BoneHeight* was considered important, but was omitted after preliminary test showed its inclusion led to unstable estimates in the analysis. This may indicate for a selective bias in the choice of the surgical procedure chosen for the patients. Furthermore, the fact that inclusion of bone-height lead to destabilization of the numerical procedure is a likely indicator that this grouping leads to highly heterogenous combinations in correlating confounding factors.

**Table 1:** Implant parameter descriptors.

**Parameter**	**Levels**	**Complete**	**Implants**	**Used**
Approach	lateral_window, transcrestal	138	16268	Yes
StudyType	CT_RCT, RS_CS	138	16268	No
Placement	1stage, 2stage, either	138	16268	No
GraftMat	autogenous, bone_substitute, either, combination, no_graft	138	16268	Yes
Membrane	membrane, no_membrane	132	14766	Yes
Implant	either, pressfit, screw	124	11974	Yes
Surface	either, machined, rough	117	11674	No
BoneHeight	lesser_5mm, greater_5mm	100	10451	No

The table shows the different implant parameter discriptors. Approach describes the use of either lateral window or transcrestal approach; *StudyType* categorizes the studies into two groups: high quality studies represented by randomized control trials (RCT) and clinical trials (CT) and studies of low quality such as case series (CS) and retrospective studies (RS). *Placement* evaluates the time point of implantation. *GraftMat* analyses possible application and type of grafting material (*either*: bone substitute or autogenous bone; *combination*: bone substitute and autogenous bone. Distribution is shown in Table 2, *Special*). *Membrane* differentiates studies using membranes from nonusers. *Implant* and *Surface* classify the type of implant and its surface and *BoneHeight* classifies the studies with bone height of less or more than 5mm residual bone prior implantation. The column *Used* describes the use of predictive parameter in modelling.

**Table 2:** Publications and references included in meta-analysis.

**Short_Pub**	**Year**	**Reference / n Implants**	**Publication**	**Special**
acocella	2011	(3, n=31)	Acocella et al.	
agamy	2010	(10, n=47)	Agamy et al.	
anitua	2009	(3, n=43)	Anitua et al.	
bae	2010	(5, n=32)	Bae et al.	
barone08	2008	(24, n=12)	Barone et al.	
barone11	2011	(5, n=201)	Barone et al.	
bassil	2011	(3, n=49)	Bassil et al.	
bergh	1998	(4, n=161)	Van den Bergh et al.	
bernardello	2011	(11, n=134)	Bernardello et al.	
blus	2008	(12, n=117)	Blus et al.	
bornstein	2008	(5, n=111)	Bornstein et al.	
bystedt	2009	(3, n=23)	Bystedt et al.	
canniza	2007	a (19, n=52), b (19, n=52)	Cannizzaro et al.	
canniza09	2009	a (5, n=44), b (6, n=38)	Cannizzaro et al.	
canullo	2012	(3, n=67)	Canullo et al.	
caubet	2011	(5, n=65)	Caubet et al.	
chaushu	2009	(2, n=72)	Chaushu et al.	
chen	2007	(9, n=47)	Chen et al.	
coatoam	1997	(20, n=85)	Coatoam et al.	Implant: 71 screw, 18 pressfit
cordioli	2001	(5, n=27)	Cordioli et al.	
crespi	2010	(13, n=30)	Crespi et al.	
cricchio	2011	(9, n=189)	Cricchio et al.	
dasmah	2012	(2, n=40)	Dasmah et al.	
deporter00	2000	(16, n=26)	Deporter et al.	
deporter05	2005	(16, n=103)	Deporter et al.	
diss	2008	(24, n=35)	Diss et al.	
engelke	2001	(7, n=44)	Engelke	
esposito	2011	(2, n=38)	Esposito et al.	
fermergard	2012	(10, n=53)	Fermergard et al.	
ferrigno	2006	(6, n=587)	Ferrigno et al.	Graftmat: almost all autogenous, very rarely combination
fugazot02	2002	(13, n=137)	Fugazzotto et al.	
fugazot202	2002	(21, n=83)	Fugazotto et al.	
galindo07	2007	(5, n=263)	Galindo-Moreno et al.	
galindo10	2010	(5, n=45)	Galindo-Moreno et al.	
guerrero	2012	(2, n=84)	Guerrero et al.	
hallman	2002	(7, n=74)	Hallman et al.	
hansen	2011	(12, n=58)	Hansen et al.	Graftmat: 29 autogenous, 29 combination
heinemann	2009	(2, n=37)	Heinemann et al.	
herzberg	2006	(14, n=203)	Herzberg et al.	Graftmat: 48 autogenous, 13 bone_substitute, 151 combination
hu	2009	(13, n=25)	Hu et al.	
irinakis	2011	(2, n=49)	Irinakis et al.	
johansso10	2010	(4, n=81)	Johansson et al. (B)	
johansso99	1999	(4, n=110)	Johansson et al. (LA)	
jurisic	2008	a (3, n=15), b (3, n=25), c (13, n=40)	Jurisic et al.	
kahnberg01	2001	(4, n=71)	Kahnberg et al.	
kahnberg08	2008	(4, n=153)	Kahnberg et al.	
kahnberg11	2011	(4, n=20)	Kahnberg et al.	
kaneko	2012	(9, n=21)	Kaneko et al.	
keller	1994	(4, n=65)	Keller et al.	
kermalli	2008	b (16, n=29), c (13, n=28)	Kermalli et al.	
kim	2011	(2, n=35)	Kim et al.	
krenmair	2007	a (5, n=28), b (5, n=12), c (13, n=14)	Krennmair et al.	
krenmair08	2008	(7, n=79)	Krennmair et al.	
lambert	2010	(2, n=102)	Lambert et al.	
lambrecht	2003	(12, n=36)	Lambrecht et al.	
leblebicio	2005	(10, n=73)	Leblebicioglu et al.	
lee	2008	(7, n=97)	Lee et al. (CY)	
leechen	2012	(2, n=12)	Lee et al. (DZ)	
leick	2005	(2, n=300)	Leick et al.	
lin	2011	(9, n=80)	Lin et al.	
lindgren	2012	(2, n=47)	Lindgren et al.	
lundgren	2004	(9, n=19)	Lundgren et al.	
maioran00	2000	(7, n=30)	Maiorano et al.	
maioran05	2005	(2, n=36)	Maiorano et al.	
mangano03	2003	(3, n=28)	Mangano et al.	
mangano07	2007	(3, n=100)	Mangano et al.	
marchet	2007	a (7, n=32), b (7, n=108)	Marchetti et al.	
markovic	2011	(10, n=40)	Markovic et al.	
mazor00	2000	(25, n=26)	Mazor et al.	
mazor99	1999	(8, n=10)	Mazor et al.	
minichetti	2008	(2, n=136)	Minichetti et al.	
nedir	2010	(10, n=24)	Nedir et al.	
peleg06	2006	(1, n=2117)	Peleg et al.	Implant: 1374 screw, 758 pressfit
peleg98	1998	(8, n=55)	Peleg et al.	
peleg99	1999	(8, n=160)	Peleg et al.	
pelegetal	1999	(8, n=57)	Peleg et al.	
pieri	2012	(5, n=90)	Pieri et al.	
pjetursson	2009	(11, n=252)	Pjetursson et al.	Graftmat: 35% bone_substitute, 65% no_graft
rodriguez	2003	(23, n=70)	Rodriguez et al.	
sakka	2011	(15, n=77)	Sakka et al.	
sbord11	2011	a (15, n=136), b (2, n=146)	Sbordone et al.	
scarano	2010	(3, n=264)	Scarano et al.	
schleier06	2006	(10, n=49)	Schleier et al.	
schleier08	2008	(10, n=59)	Schleier et al.	
sforza	2008	(22, n=39)	Sforza et al.	
siervo	2004	(7, n=72)	Siervo et al.	
simonpieri	2011	(2, n=52)	Simonpieri et al.	
sohn	2011	(18, n=113)	Sohn et al.	Implant: 109 screw, 4 pressfit
stavropoul	2007	(22, n=33)	Stavropoulos et al.	
stricker	2003	(4, n=180)	Stricker et al.	
sungcho	2008	(12, n=130)	Lee et al. (JH)	
thor	2007	(9, n=44)	Thor et al.	
torres	2009	a (3, n=153), b (3, n=129)	Torres et al.	
vicente	2010	a (5, n=22), b (5, n=68)	De Vicente et al.	
viscioni	2010	(3, n=64)	Viscioni et al.	
voss	2010	a (4, n=118), b (3, n=168)	Voss et al.	
wannfors	2000	(4, n=132)	Wannfors et al.	
watzek	1998	(17, n=142)	Watzek et al.	Graftmat: 49 autogenous, 16 bone_substitute, 70 combination
winter	2002	(10, n=54)	Winter et al.	
yamada	2008	(3, n=41)	Yamada et al.	
yamamich	2008	(2, n=159)	Yamamichi et al.	
zetterqvis	2004	(7, n=64)	Hallman et al.	
zijderveld	2005	(14, n=41)	Zijderveld et al.	Graftmat: 15 autogenous, 26 bone_substitute
zinner	1996	(8, n=212)	Zinner et al.	
zitzman	1998	a (2, n=7), b (2, n=13)	Zitzmann et al.	
zitzmann98	1998	(6, n=57)	Zitzmann et al.	

The table shows the publications and the according references used in this meta-analysis (Table S1). In column Reference, the first number in brackets refers to the row number (Combination) in table 3 that gives the combination of parameters for this reference. In case of multiple references in one publication, marker letters a, b, c were added, e.g the study Cannizzaro et al. has references to 2 combinations a and b which both happen to refer to row 19 in table 3 because they only differ by parameter Placement which is not used in this meta-analysis. Publications with simplifiying assumptions due to incomplete information are specified in column Special.

**Table 3:** Combinations of parameters.

**Combination**	**Membrane**	**Approach**	**Implant**	**GraftMat**	**n**
1	membrane	lateral_window	either	either	2117
2	membrane	lateral_window	screw	bone_substitute	1365
3	no_membrane	lateral_window	screw	bone_substitute	1150
4	no_membrane	lateral_window	screw	autogenous	1141
5	membrane	lateral_window	screw	combination	1008
6	no_membrane	transcrestal	screw	autogenous	682
7	no_membrane	lateral_window	screw	combination	600
8	membrane	lateral_window	pressfit	combination	494
9	no_membrane	lateral_window	screw	no_graft	400
10	no_membrane	transcrestal	screw	no_graft	399
11	no_membrane	transcrestal	screw	either	386
12	membrane	lateral_window	screw	either	341
13	no_membrane	transcrestal	screw	bone_substitute	275
14	no_membrane	lateral_window	screw	either	244
15	membrane	lateral_window	screw	autogenous	213
16	no_membrane	transcrestal	pressfit	bone_substitute	157
17	no_membrane	lateral_window	pressfit	either	142
18	no_membrane	lateral_window	either	bone_substitute	113
19	membrane	lateral_window	pressfit	autogenous	104
20	no_membrane	transcrestal	either	combination	85
21	membrane	transcrestal	screw	autogenous	83
22	no_membrane	transcrestal	screw	combination	72
23	no_membrane	lateral_window	pressfit	bone_substitute	70
24	membrane	transcrestal	screw	bone_substitute	47
25	membrane	lateral_window	pressfit	bone_substitute	26

The table shows the combination of parameters used in meta-analysis and the number of implants for each case, sorted by number of implants descending. Column Combination is referenced from column Reference in table 2.

A final set of 106 publications, with 119 references (i.e. groups with distinct treatments within publications) and 11714 implants was included in this meta-analysis (Table 2).

### Univariate Kaplan-Meier analysis

**Figure 2:**
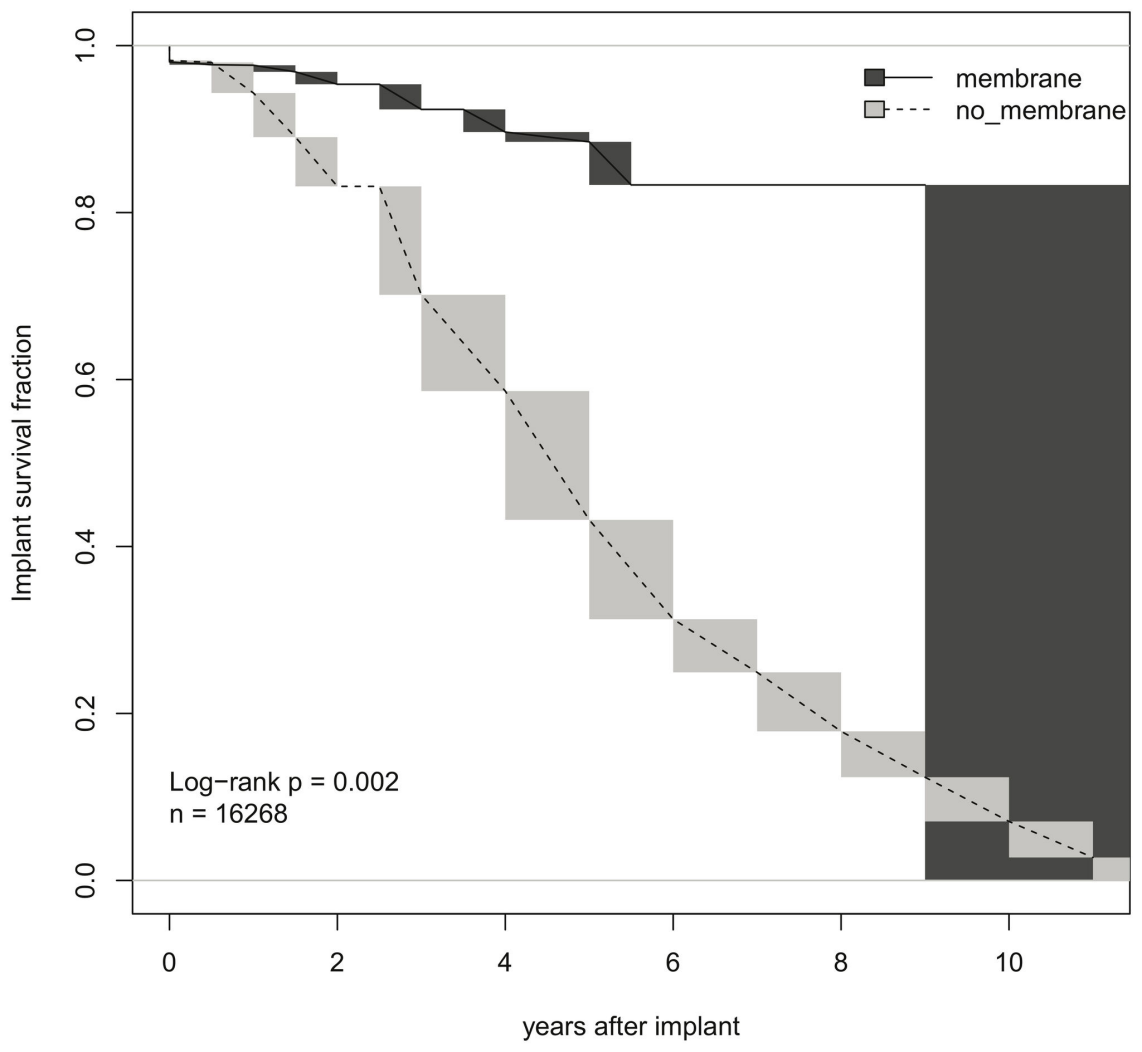
Univariate Kaplan-Meier analysis plots for interval-censored data and subset of publications used in this meta-analysis. The gray bars show regions of indetermination from the maximum likelihood estimation of interval-censored data (Turnbull, 1976).

Kaplan-Meier plots for interval-censored data were grouped by the use of membranes (Figure 2). The gray bars show regions of indetermination from the maximum likelihood estimation of interval-censored data [52]. The prolonged implant survival for the membrane-group is highly significant (p=0.002). However, the univariate analysis is misleading and given here as an illustration only, because it assumes that other factors were kept constant or at least do not co-vary strongly with the use of membranes. To correct for such correlations, a multivariate analysis was performed.

### Multivariate interval-censored Cox regression

**Figure 3:**
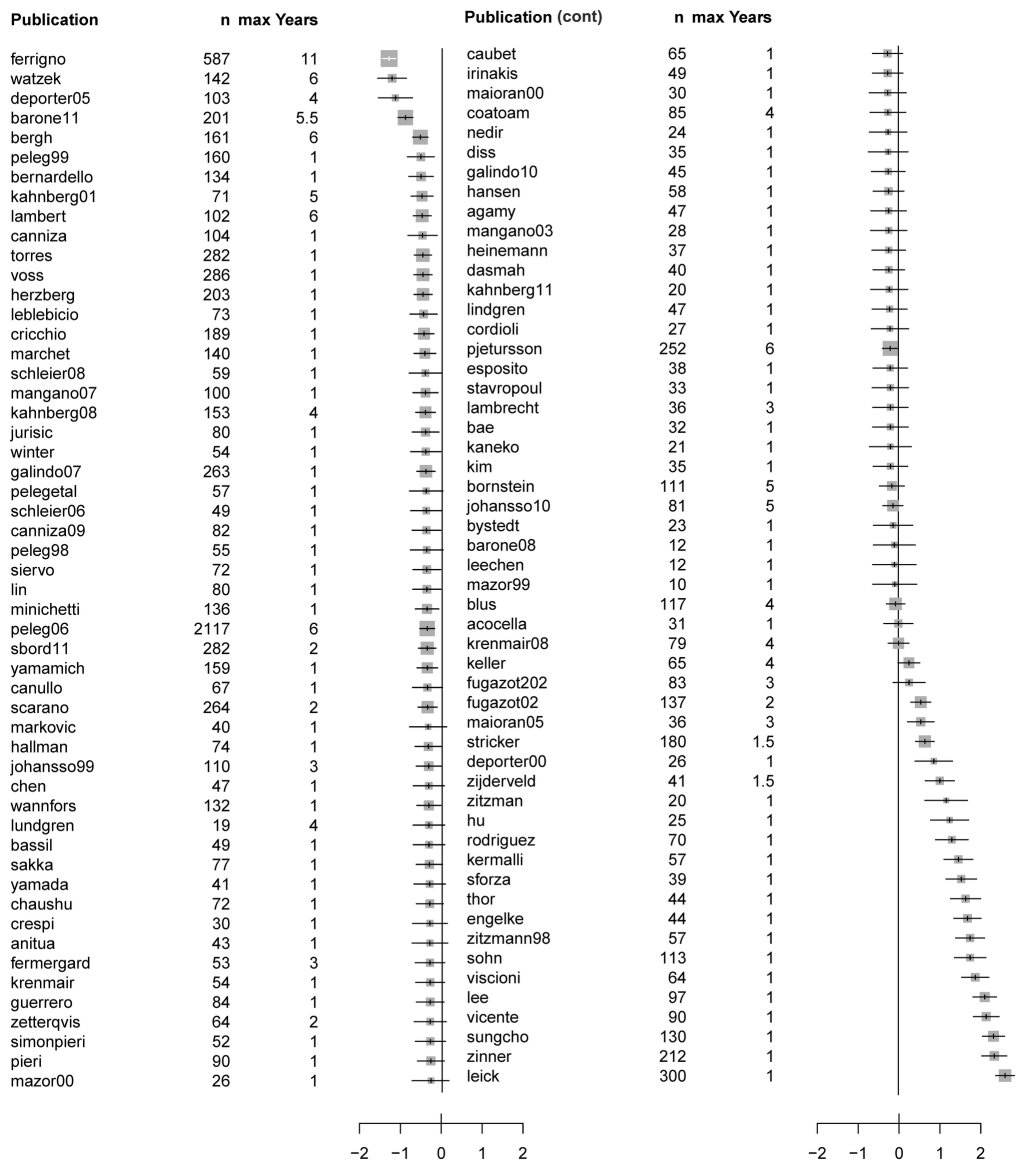
Forest plot of log hazard ratio with 95% confidence intervals for publications in meta-analysis. n: number of implants, max Years: maximal follow-up time.

The effects of the different study groups were factored giving log hazard ratios for all publications in this meta-analysis (Figure 3). To conserve the power of planned cross-over studies, the figures do not use the references to subgroups, i.e. when two treatment combinations are reported in one publication, they are combined. The baseline-hazard was computed over the range of up to 6 years after implantation; since most studies have follow-up times of 1 year, the modelling algorithm has to make assumptions about the future survival for short records. This leads to extreme hazard ratios for the publications with short follow-up times that occur at the bottom of the list; studies with long follow-up times have smaller hazard ratios.

### Method effect

**Figure 4:**
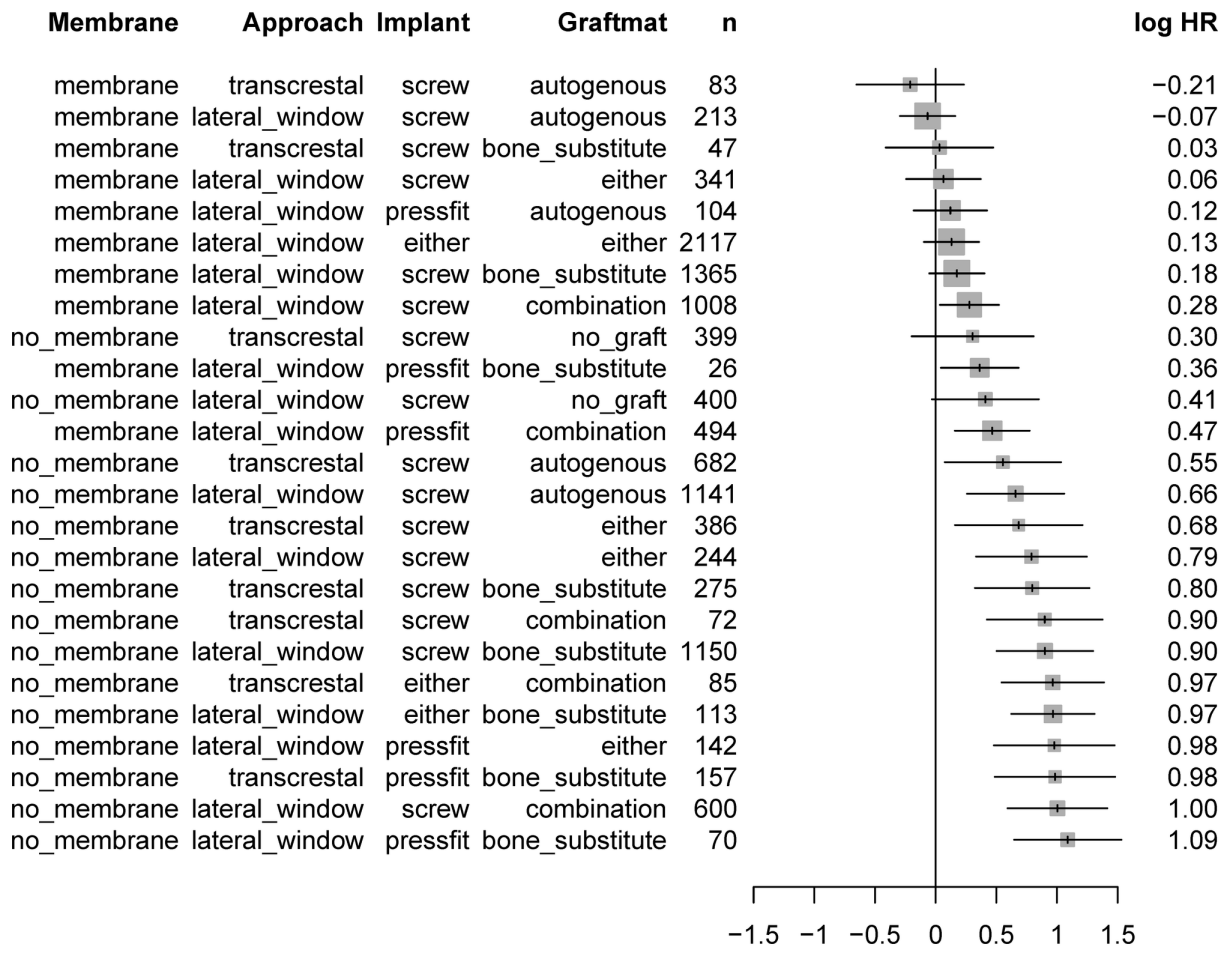
Forest plot of logarithmic hazard ratios (HR) and their 95%-confidence intervals for all method combinations. n: number of implants. Low values at the top represent longer implant survival.

**Figure 5:**
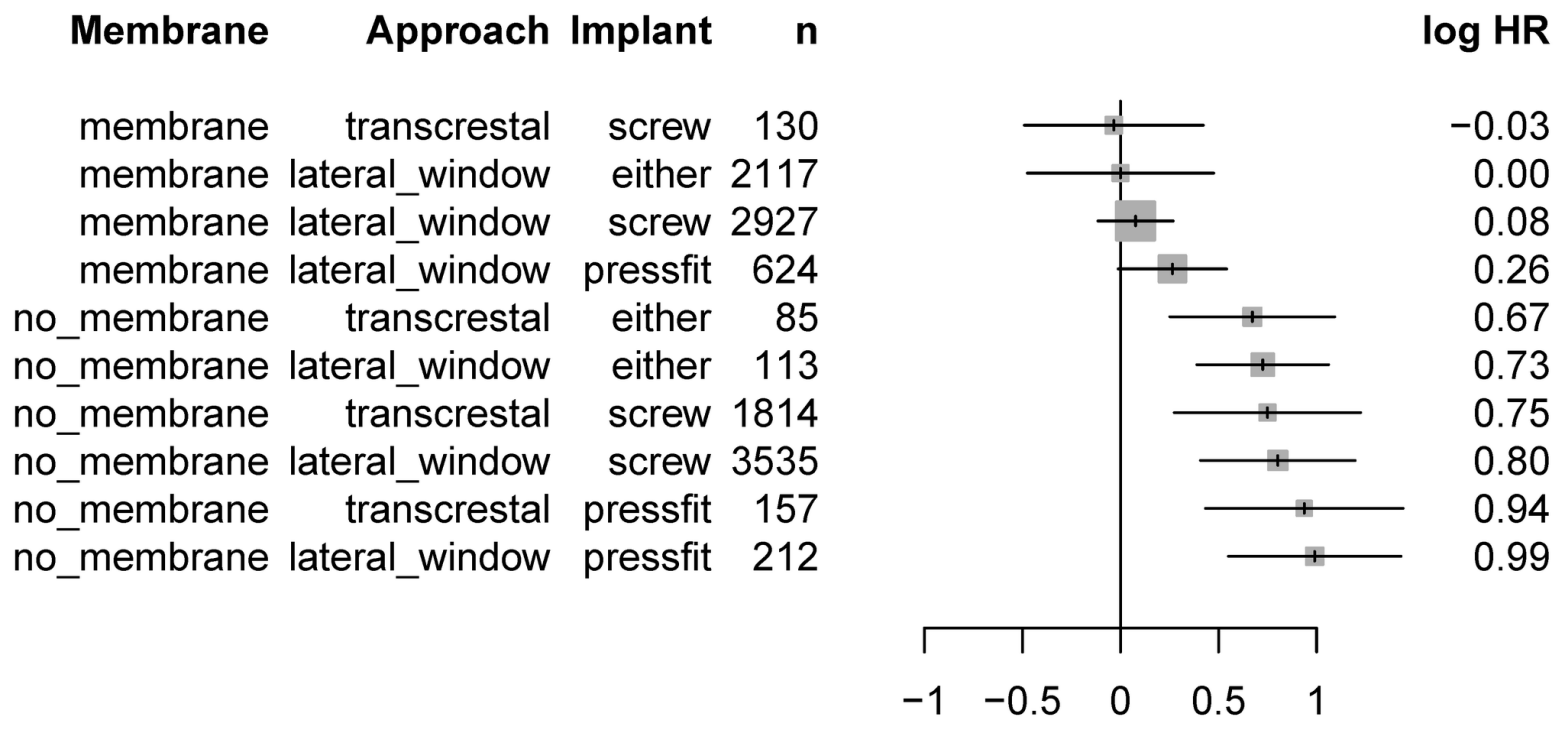
Log hazard ratios (HR) and their 95%-confidence intervals, with simplified model omitting parameter *GraftMat*. Absolute values of HR should not be compared with those in Figure 4 because reference values or HR are not comparable. n: number of implants.

Results ranged from -0.21 to 1.09 of the logarithmic hazard ratio, from best to worst, which corresponds to a range of the hazard ratio of 3.7 (Figure 4). The most evident feature was the clustering of methods with membrane at the top of the list. The arrangement of the four levels of grafting material (*GraftMat*) was effectively random. To clarify the overall picture, the model was recomputed by excluding this parameter entirely (Figure 5). This simplified model corroborated that solely combinations including membranes remained at the top of the list. Furthermore, a strong increase in the hazard ratio of the no_membrane block from 0.26 to 0.67 was observed. Even in the simplified model, there was no evidence for any preference for parameters Approach and Implant.

### Hazard functions

**Figure 6:**
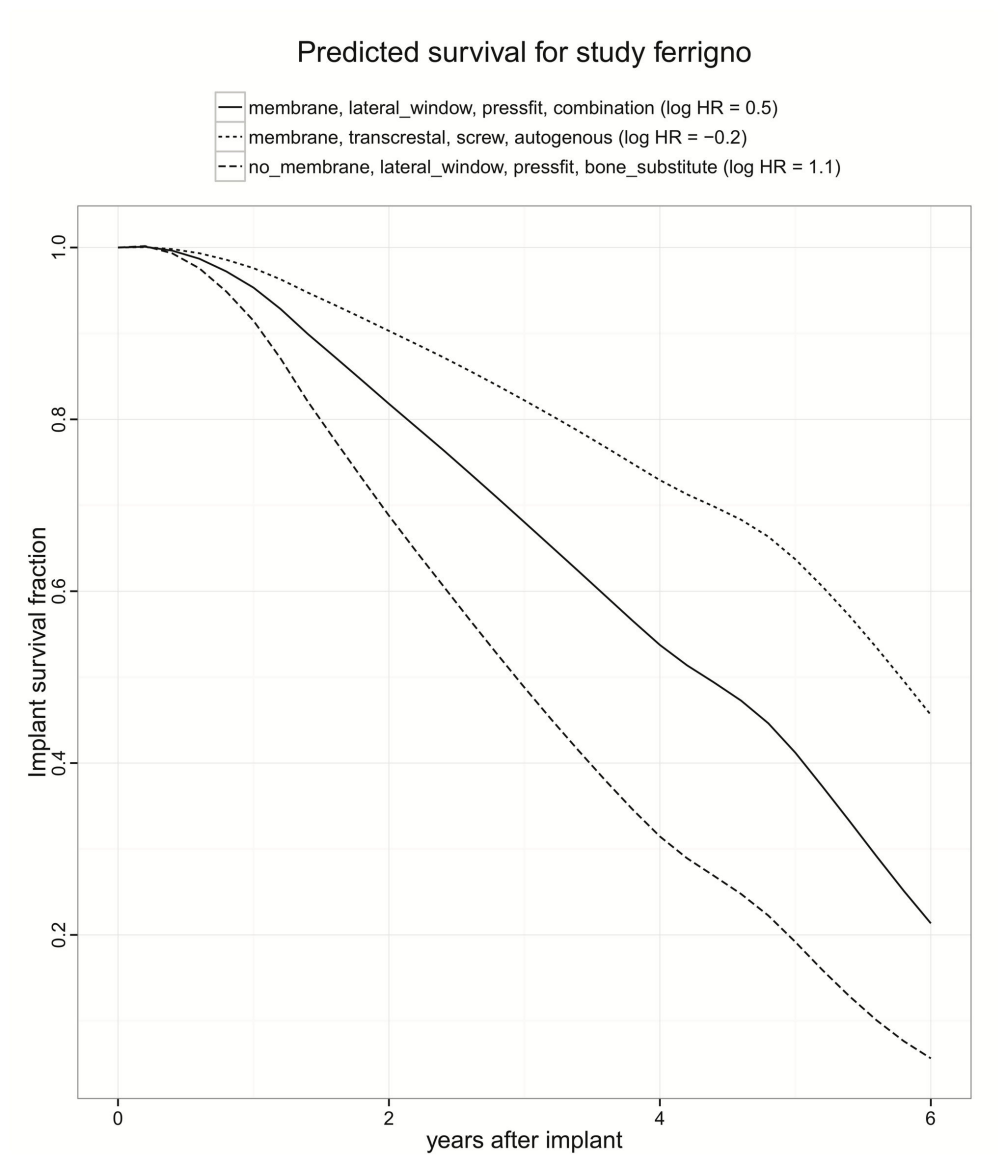
Three samples of implant survival curves, estimated from a Cox regression model for interval-censored data. The curves are similar, and less extreme than the mono-variable survival plot; this is a feature of the Cox regression, where only the hazard ratio determines the degree of stretching. Note that this is not an average curve, but a hypothetical prediction for one study with long observation time.

To illustrate survival curves, three samples are shown, covering the range from smallest to larges hazard ratio (Figure 6). The curves are similar, and less extreme than the mono-variable survival plot; this is a feature of the Cox regression, where only one number, the hazard ratio, determines the degree of stretching. Note that this is not an average curve, but a hypothetical prediction for one study with long observation time; giving a population average is not meaningful for studies with a large range. The Cox model might be wrong in some aspects, but the simplifications are the precondition for a successful covariate analysis. However, because of the scarcity of data for post-implantation follow-up visits after 3 years, it is questionably if the change in slope after three years is real or biased by selective dropouts of studies.

## Discussion

To date, the influence of treatment modalities on survival and success rates of endosseous implants placed in the grafted maxillary sinus is not yet deciphered [6,17,53]. Accordingly, evidence-based decision-making on how to treat the patient at best possible conditions is still impaired. To address this problem the present systematic review and meta-analysis investigated the role of different factor combinations and associated reciprocal effects in sinus augmentation and successful implantation procedures. Previous meta-analyses were substantially affected by the inconsistency of study designs. Concurrently causative unsuitable statistical analysis accounted for the low clinical evidence [54,55]. Statistical methods such as multivariable Poisson regression, life table modelling and basic univariate Kaplan-Meier Analysis are a commonly used to assess implant survival and success rates. Still, this type of analysis does not take the influence of other factors or covariables such as study effects into account. To cite an example, the preliminary conducted univariate analysis on implant success indicated, in concert with recent investigations [6], that the use of membranes is highly significant. However, if membranes would be preferably used in combination with screws and autogenous material, and non-membranes preferably with press-fit implants, and bone-substitute materials the difference between the membrane groups could equally well be attributed to the effect of the implant and the graft material. Hence, meta-analysis must correct for study effects to obtain publication-independent estimates of method effects.

To address such study effects our analytical approach was to conduct survival analysis using multivariate interval-censored Cox regression to specifically analyse method effects and corresponding hazard ratios. This modelling could correct for the fact that studies with only 25 of the 32 possible combinations were available, and that some combinations were represented only by one study. In Cox-regression [56], first a common non-parametric baseline survival curve is estimated; for each combination of parameters, only one parameter, the hazard ratio, quantifies how the survival curve is stretched or compressed compared to the baseline curve [57]. The applied Cox model is a simplification and, for example, does not allow for the crossing of survival curves. Still, simplification is the precondition for a successful covariate analysis that enables the estimation of covariable effects when not all combinations are known, which was impossible under the more general Kaplan-Meier assumption. To illustrate the basic (baseline) log hazard ratio of implant survival for each publication the different methods have been factored out to correct for study effects. Accordingly, the presented hazard ratios should be recognized as a correction to the hazard one would expect from the methods used in the publication. For example, if success with all methods improved over time, this would lead to lower hazards in more recent publications, and by including the study as a covariable in the analysis one would implicitly correct for this trend.

Notably, the total number of implants investigated in the publications had no direct effect on the basic (baseline) log hazard ratio, whereas, follow-up time of each publication emerged to be the most prominent factor. This may advocate for a prolonged follow-up time to safely assess valid implant survival in consideration of co-factors [17] such as technical complications with supra-constructions, especially screw or abutment loosening [58], marginal bone loss [59], smoking or oral hygiene [60,61]. However, the time frame of one year follow-up is prevalently used to assess implant success, predominantly the effective osseointegration of the implant irrespective of the type of functional loading [62-64]. Implant loss in the first year was found low, consequently the main information was obtained from the few studies with follow-up time between 2 years and 7 years; this selective drop-out is the most critical factor affecting the reliability of the results of this meta-analysis. Taken together it is advisable to avoid rating the quality of individual studies, but rather to consider inclusion of study effects as an overall compensation to factor out random study variations from method effects.

To decipher the hazard ratio for all methods combinations, as predicted from a model that corrects for the publication effect, was the primary endpoint of this meta-analysis. Results showed a pronounced clustering of methods that included membranes at the top of the list. None of the other columns exhibited such an asymmetry in distribution. This highlights the paramount importance of the use of membranes, independent of other details and corroborates the finding of the simpler univariate Kaplan Meier analysis. Moreover, this meta-analysis revealed a notable step in the hazard ratio between the membrane and the no_membrane group from 0.26 to 0.67, presenting an even stronger indicator of the fact that the use of membranes creates a different league. Interestingly, the arrangement of the four levels of grafting material (*GraftMat*) was effectively random and thus the model was recomputed excluding this parameter entirely. In this simplified model combinations using membranes were still completely at the top. Furthermore, there was no evidence for any preference for parameters Approach and Implant. Moreover, the treatment parameters surgical approach, grafting material, and implant type showed no selective preference. Recent investigations concluded similar results with the prognosis of implants unaffected by the type of graft material, residual bone height and time of implant placement [65]. Other investigations advocated that the use of autogenous bone, rough and screw-type implants as well as membranes exhibit favourable results [6]. However, combinations of solely 2 factors were statistically admissible within the limitations of life table modelling. In comparison, implant survival curves, estimated from the present Cox regression model for interval-censored data, showed the lowest hazard ratio for the combination of membrane, screw-type implant, transcrestal approach, and autogenous bone. Yet, data were interval censored and the time of event only known to be within a given range determined by the inter-visit interval. However, because of the scarcity of data for post-implantation follow-up visits after 3 years, it is questionably if the change in slope after three years is real or biased by selective dropouts of studies. In summary, with randomized designs being ethically unacceptable more complete information from split-mouth designs is crucial to negotiate prospective confounding and further improve clinical evidence.

## Conclusion

Other than conventional statistical methods the Bayesian multivariate interval-censored Cox regression helps to take covariables such as study effects into account. While the present meta-analysis does not give evidence for any significant method effect besides that of membrane application, this dominance should be interpreted in the light of the correction for study-group effects factored out in the meta-analysis. It cannot be excluded that other factors in use by a team experienced with this combination might still be relevant for implant survival, because team and method effects are confounded. Consequently, more studies on follow-up visits exceeding 3 years are needed to address confounding and improve clinical evidence. Nevertheless, dominance of the membrane effect means that membranes extent implant survival independently of the surgeons’ special preferences and skills and thus may improve future decision-making in dento-maxillary rehabilitation.

## Supporting Information

Checklist S1 **PRISMA Checklist.** The PRISMA Checklist documents the methods of analysis and inclusion criteria that were specified in advance by the reviewers according to PRISMA guidelines.
(DOC)

Table S1 **Reference appendix.** The table documents the full reference for the abbreviated publications listed in table 2.
(DOCX)
