# Supplementary material for: Long-Term Survival of Dental Implants Placed in the Grafted Maxillary Sinus: Systematic Review and Meta-Analysis of Treatment Modalities
Source: PLoS One. 2013 Sep 18;8(9):e75357. doi: 10.1371/journal.pone.0075357 (PMC3776785; doi:10.1371/journal.pone.0075357)
Supplement: Checklist S1 — PRISMA Checklist. The PRISMA Checklist documents the methods of analysis and inclusion criteria that were specified in advance by the reviewers according to PRISMA guidelines. (DOC) [file pone.0075357.s001.doc]

| **Section/topic** | **#** | **Checklist item** | **Reported in section** |
| --- | --- | --- | --- |
| **TITLE** | | |  |
| Title | 1 | Identify the report as a systematic review, meta-analysis, or both. | Titlepage |
| **ABSTRACT** | | |  |
| Structured summary | 2 | Provide a structured summary including, as applicable: background; objectives; data sources; study eligibility criteria, participants, and interventions; study appraisal and synthesis methods; results; limitations; conclusions and implications of key findings; systematic review registration number. | Abstract |
| **INTRODUCTION** | | |  |
| Rationale | 3 | Describe the rationale for the review in the context of what is already known. | Introduction |
| Objectives | 4 | Provide an explicit statement of questions being addressed with reference to participants, interventions, comparisons, outcomes, and study design (PICOS). | Introduction,  PICOS Appendix |
| **METHODS** | | |  |
| Protocol and registration | 5 | Indicate if a review protocol exists, if and where it can be accessed (e.g., Web address), and, if available, provide registration information including registration number. | Materials and Methods (M&M) |
| Eligibility criteria | 6 | Specify study characteristics (e.g., PICOS, length of follow-up) and report characteristics (e.g., years considered, language, publication status) used as criteria for eligibility, giving rationale. | M&M &  PICOS Appendix |
| Information sources | 7 | Describe all information sources (e.g., databases with dates of coverage, contact with study authors to identify additional studies) in the search and date last searched. | M&M |
| Search | 8 | Present full electronic search strategy for at least one database, including any limits used, such that it could be repeated. | M&M |
| Study selection | 9 | State the process for selecting studies (i.e., screening, eligibility, included in systematic review, and, if applicable, included in the meta-analysis). | M&M |
| Data collection process | 10 | Describe method of data extraction from reports (e.g., piloted forms, independently, in duplicate) and any processes for obtaining and confirming data from investigators. | M&M |
| Data items | 11 | List and define all variables for which data were sought (e.g., PICOS, funding sources) and any assumptions and simplifications made. | M&M &  PICOS Appendix |

| **Section/topic** | **#** | **Checklist item** | **Reported on page #** |
| --- | --- | --- | --- |
| Risk of bias in individual studies | 12 | Describe methods used for assessing risk of bias of individual studies (including specification of whether this was done at the study or outcome level), and how this information is to be used in any data synthesis. | Appendix1 |
| Summary measures | 13 | State the principal summary measures (e.g., risk ratio, difference in means). | M&M |
| Synthesis of results | 14 | Describe the methods of handling data and combining results of studies, if done, including measures of consistency (e.g., I^2^) for each meta-analysis. | M&M |
| Risk of bias across studies | 15 | Specify any assessment of risk of bias that may affect the cumulative evidence (e.g., publication bias, selective reporting within studies). | Appendix2 |
| Additional analyses | 16 | Describe methods of additional analyses (e.g., sensitivity or subgroup analyses, meta-regression), if done, indicating which were pre-specified. | M&M |
| **RESULTS** | | |  |
| Study selection | 17 | Give numbers of studies screened, assessed for eligibility, and included in the review, with reasons for exclusions at each stage, ideally with a flow diagram. | Results |
| Study characteristics | 18 | For each study, present characteristics for which data were extracted (e.g., study size, PICOS, follow-up period) and provide the citations. | Table 2 |
| Risk of bias within studies | 19 | Present data on risk of bias of each study and, if available, any outcome level assessment (see item 12). | Table 2 |
| Results of individual studies | 20 | For all outcomes considered (benefits or harms), present, for each study: (a) simple summary data for each intervention group (b) effect estimates and confidence intervals, ideally with a forest plot. | Table 2 |
| Synthesis of results | 21 | Present results of each meta-analysis done, including confidence intervals and measures of consistency. | Figure 2 |
| Risk of bias across studies | 22 | Present results of any assessment of risk of bias across studies (see Item 15). | Table 2, Appendix1 |
| Additional analysis | 23 | Give results of additional analyses, if done (e.g., sensitivity or subgroup analyses, meta-regression [see Item 16]). | Table 1,  Figure 1, Results |
| **DISCUSSION** | | |  |
| Summary of evidence | 24 | Summarize the main findings including the strength of evidence for each main outcome; consider their relevance to key groups (e.g., healthcare providers, users, and policy makers). | Results |
| Limitations | 25 | Discuss limitations at study and outcome level (e.g., risk of bias), and at review-level (e.g., incomplete retrieval of identified research, reporting bias). | Results |
| Conclusions | 26 | Provide a general interpretation of the results in the context of other evidence, and implications for future research. | Results |
| **FUNDING** | | |  |
| Funding | 27 | Describe sources of funding for the systematic review and other support (e.g., supply of data); role of funders for the systematic review. | none |

**PICOS Appendix:**

**PICOS Statement**

**Population:** There were no limitations or precise definition of a patient population. All patients meeting the intervention criteria were considered eligible.

**Intervention:** single intervention sinus augmentation with root-form implants and a minimum of 6-month follow-up time.

**Comparator:** Objective of the present meta-analysis is to compare covariables (*Approach*, *StudyType, Placement*, *GraftMat, Membrane, Implant, Surface and BoneHeight)* and their combinations with each other (Table 1).

**Outcome:** Results are given as log-hazard ratios (HR) relative to a median baseline hazard and illustrated as forest plots and survival curves.

**Study Design:** Case series, case control studies, cohort studies and randomized controlled trials were considered eligible.

**Appendix 1:**

About 1/3 of the studies/publications had multiple branches, e.g. with/without membrane. Pairing information within-study was use in a Bayesian design to correct for bias of individual studies; this approach is similar to a design using frailties or a mixed-model in the classical framework. Details are describe in the methods section "Multivariate analysis used a Bayesian approach as implemented in package dynsurv (49) to separate the effect of publication from that of the implantation methods".

**Appendix 2:**

However, because of the scarcity of data for post-implantation follow-up visits after 3 years, it is questionably if the change in slope after three years is real or biased by selective dropouts of studies. (Zufügen:) A bias due by study-selection in the long-term follow-up cannot be ruled out with

currently available data.

*From:*  Moher D, Liberati A, Tetzlaff J, Altman DG, The PRISMA Group (2009). *P*referred *R*eporting *I*tems for *S*ystematic Reviews and *M*eta-*A*nalyses: The PRISMA Statement. PLoS Med 6(6): e1000097. doi:10.1371/journal.pmed1000097

**For more information, visit** [**www.prisma-statement.org**](http://www.consort-statement.org/)**.**
