# Supplementary material for: Long-Term Survival of Dental Implants Placed in the Grafted Maxillary Sinus: Systematic Review and Meta-Analysis of Treatment Modalities
Source: PLoS One. 2013 Sep 18;8(9):e75357. doi: 10.1371/journal.pone.0075357 (PMC3776785; doi:10.1371/journal.pone.0075357)
Supplement: Table S1 — Reference appendix. The table documents the full reference for the abbreviated publications listed in table 2. (DOCX) [file pone.0075357.s002.docx]

**Table S1.** Reference appendix

Publication [1-106]

1. Acocella A, Bertolai R, Nissan J, Sacco R (2011) Clinical, histological and histomorphometrical study of maxillary sinus augmentation using cortico-cancellous fresh frozen bone chips. J Craniomaxillofac Surg 39: 192–199.

2. Agamy EMTM, Niedermeier W (2010) Indirect sinus floor elevation for osseointegrated prostheses. A 10-year prospective study. J Oral Implantol 36: 113–121.

3. Anitua E, Prado R, Orive G (2009) A lateral approach for sinus elevation using PRGF technology. Clinical Implant Dentistry and Related Research 11 Suppl 1: e23–e31.

4. Bae J-H, Kim Y-K, Kim S-G, Yun P-Y, Kim J-S (2010) Sinus bone graft using new alloplastic bone graft material (Osteon)-II: clinical evaluation. Oral Surg Oral Med Oral Pathol Oral Radiol Endod 109: e14–e20.

5. Barone A, Cornelini R, Ciaglia R, Covani U (2008) Implant placement in fresh extraction sockets and simultaneous osteotome sinus floor elevation: a case series. Int J Periodontics Restorative Dent 28: 283–289.

6. Barone A, Orlando B, Tonelli P, Covani U (2011) Survival rate for implants placed in the posterior maxilla with and without sinus augmentation: a comparative cohort study. Journal of Periodontology 82: 219–226.

7. Bassil J, Senni K, Changotade S, Baroukh B, Kassis C, et al. (2011) Expression of MMP-2, 9 and 13 in newly formed bone after sinus augmentation using inorganic bovine bone in human. J Periodont Res 46: 756–762.

8. van den Bergh JP, Bruggenkate ten CM, Krekeler G, Tuinzing DB (1998) Sinusfloor elevation and grafting with autogenous iliac crest bone. Clinical Oral Implants Research 9: 429–435.

9. Bernardello F, Righi D, Cosci F, Bozzoli P, Soardi CM, et al. (2011) Crestal sinus lift with sequential drills and simultaneous implant placement in sites with. Implant Dent 20: 439–444.

10. Blus C, Szmukler-Moncler S, Salama M, Salama H, Garber D (2008) Sinus bone grafting procedures using ultrasonic bone surgery: 5-year experience. Int J Periodontics Restorative Dent 28: 221–229.

11. Bornstein MM, Chappuis V, Arx von T, Buser D (2008) Performance of dental implants after staged sinus floor elevation procedures: 5-year results of a prospective study in partially edentulous patients. Clinical Oral Implants Research 19: 1034–1043.

12. Bystedt H, Rasmusson L (2009) Porous titanium granules used as osteoconductive material for sinus floor augmentation: a clinical pilot study. Clinical Implant Dentistry and Related Research 11: 101–105.

13. Cannizzaro G, Leone M, Consolo U, Ferri V, Licitra G, et al. (2007) Augmentation of the posterior atrophic edentulous maxilla with implants placed in the ulna: a prospective single-blind controlled clinical trial. Int J Oral Maxillofac Implants 22: 280–288.

14. Cannizzaro G, Felice P, Leone M, Viola P, Esposito M (2009) Early loading of implants in the atrophic posterior maxilla: lateral sinus lift with autogenous bone and Bio-Oss versus crestal mini sinus lift and 8-mm hydroxyapatite-coated implants. A randomised controlled clinical trial. Eur J Oral Implantol 2: 25–38.

15. Canullo L, Patacchia O, Sisti A, Heinemann F (2012) Implant restoration 3 months after one stage sinus lift surgery in severely resorbed maxillae: 2-year results of a multicenter prospective clinical study. Clinical Implant Dentistry and Related Research 14: 412–420.

16. Caubet J, Petzold C, Sáez-Torres C, Morey M, Iriarte JI, et al. (2011) Sinus graft with safescraper: 5-year results. J Oral Maxillofac Surg 69: 482–490.

17. Chaushu G, Mardinger O, Calderon S, Moses O, Nissan J (2009) The use of cancellous block allograft for sinus floor augmentation with simultaneous implant placement in the posterior atrophic maxilla. Journal of Periodontology 80: 422–428.

18. Chen T-W, Chang H-S, Leung K-W, Lai Y-L, Kao S-Y (2007) Implant placement immediately after the lateral approach of the trap door window procedure to create a maxillary sinus lift without bone grafting: a 2-year retrospective evaluation of 47 implants in 33 patients. YJOMS 65: 2324–2328.

19. Coatoam GW, Krieger JT (1997) A four-year study examining the results of indirect sinus augmentation procedures. J Oral Implantol 23: 117–127.

20. Cordioli G, Mazzocco C, Schepers E, Brugnolo E, Majzoub Z (2001) Maxillary sinus floor augmentation using bioactive glass granules and autogenous bone with simultaneous implant placement. Clinical and histological findings. Clinical Oral Implants Research 12: 270–278.

21. Crespi R, Capparè P, Gherlone E (2010) Osteotome sinus floor elevation and simultaneous implant placement in grafted biomaterial sockets: 3 years of follow-up. Journal of Periodontology 81: 344–349.

22. Cricchio G, Sennerby L, Lundgren S (2011) Sinus bone formation and implant survival after sinus membrane elevation and implant placement: a 1- to 6-year follow-up study. Clinical Oral Implants Research 22: 1200–1212.

23. Dasmah A, Hallman M, Sennerby L, Rasmusson L (2012) A clinical and histological case series study on calcium sulfate for maxillary sinus floor augmentation and delayed placement of dental implants. Clinical Implant Dentistry and Related Research 14: 259–265.

24. Deporter D, Todescan R, Caudry S (2000) Simplifying management of the posterior maxilla using short, porous-surfaced dental implants and simultaneous indirect sinus elevation. Int J Periodontics Restorative Dent 20: 476–485.

25. Deporter DA, Caudry S, Kermalli J, Adegbembo A (2005) Further data on the predictability of the indirect sinus elevation procedure used with short, sintered, porous-surfaced dental implants. Int J Periodontics Restorative Dent 25: 585–593.

26. Diss A, Dohan DM, Mouhyi J, Mahler P (2008) Osteotome sinus floor elevation using Choukroun's platelet-rich fibrin as grafting material: a 1-year prospective pilot study with microthreaded implants. Oral Surg Oral Med Oral Pathol Oral Radiol Endod 105: 572–579.

27. W E (2001) Subantroskopisch laterobasale Sinusbodenaugmentation mit Algipore: Eine klinische Studie. Zeitschrift für Zahnärztliche Implantologie XVII: 154.

28. Esposito M, Pellegrino G, Pistilli R, Felice P (2011) Rehabilitation of postrior atrophic edentulous jaws: prostheses supported by 5 mm short implants or by longer implants in augmented bone? One-year results from a pilot randomised clinical trial. Eur J Oral Implantol 4: 21–30.

29. Fermergård R, Åstrand P (2012) Osteotome sinus floor elevation without bone grafts--a 3-year retrospective study with Astra Tech implants. Clinical Implant Dentistry and Related Research 14: 198–205.

30. Ferrigno N, Laureti M, Fanali S (2006) Dental implants placement in conjunction with osteotome sinus floor elevation: a 12-year life-table analysis from a prospective study on 588 ITIRimplants. Clinical Oral Implants Research 17: 194–205.

31. Fugazzotto PA (2002) Immediate implant placement following a modified trephine/osteotome approach: success rates of 116 implants to 4 years in function. Int J Oral Maxillofac Implants 17: 113–120.

32. Fugazzotto PA, De PS (2002) Sinus floor augmentation at the time of maxillary molar extraction: success and failure rates of 137 implants in function for up to 3 years. Journal of Periodontology 73: 39–44.

33. Galindo-Moreno P, Avila G, Fernández-Barbero JE, Aguilar M, Sánchez-Fernández E, et al. (2007) Evaluation of sinus floor elevation using a composite bone graft mixture. Clinical Oral Implants Research 18: 376–382.

34. Galindo-Moreno P, Padial-Molina M, Fernández-Barbero JE, Mesa F, Rodríguez-Martínez D, et al. (2010) Optimal microvessel density from composite graft of autogenous maxillary cortical bone and anorganic bovine bone in sinus augmentation: influence of clinical variables. Clinical Oral Implants Research 21: 221–227.

35. Guerrero JS, Al-Jandan BA (2012) Allograft for maxillary sinus floor augmentation: a retrospective study of 90 cases. Implant Dent 21: 136–140.

36. Hallman M, Hedin M, Sennerby L, Lundgren S (2002) A prospective 1-year clinical and radiographic study of implants placed after maxillary sinus floor augmentation with bovine hydroxyapatite and autogenous bone. YJOMS 60: 277–285.

37. Hansen EJ, Schou S, Harder F, Hjørting-Hansen E (2011) Outcome of implant therapy involving localised lateral alveolar ridge and/or sinus floor augmentation: a clinical and radiographic retrospective 1-year study. Eur J Oral Implantol 4: 257–267.

38. Heinemann F, Mundt T, Biffar R, Gedrange T, Goetz W (2009) A 3-year clinical and radiographic study of implants placed simultaneously with maxillary sinus floor augmentations using a new nanocrystalline hydroxyapatite. J Physiol Pharmacol 60 Suppl 8: 91–97.

39. Herzberg R, Dolev E, Schwartz-Arad D (2006) Implant marginal bone loss in maxillary sinus grafts. Int J Oral Maxillofac Implants 21: 103–110.

40. Hu X, Lin Y, Metzmacher A-R, Zhang Y (2009) Sinus membrane lift using a water balloon followed by bone grafting and implant placement: a 28-case report. Int J Prosthodont 22: 243–247.

41. Irinakis T (2011) Efficacy of injectable demineralized bone matrix as graft material during sinus elevation surgery with simultaneous implant placement in the posterior maxilla: clinical evaluation of 49 sinuses. J Oral Maxillofac Surg 69: 134–141.

42. Johansson B, Wannfors K, Ekenbäck J, Smedberg JI, Hirsch J (1999) Implants and sinus-inlay bone grafts in a 1-stage procedure on severely atrophied maxillae: surgical aspects of a 3-year follow-up study. Int J Oral Maxillofac Implants 14: 811–818.

43. Johansson L-A, Isaksson S, Lindh C, Becktor JP, Sennerby L (2010) Maxillary sinus floor augmentation and simultaneous implant placement using locally harvested autogenous bone chips and bone debris: a prospective clinical study. J Oral Maxillofac Surg 68: 837–844.

44. Jurisic M, Markovic A, Radulovic M, Brkovic BMB, Sándor GKB (2008) Maxillary sinus floor augmentation: comparing osteotome with lateral window immediate and delayed implant placements. An interim report. Oral Surg Oral Med Oral Pathol Oral Radiol Endod 106: 820–827.

45. Kahnberg KE, Ekestubbe A, Gröndahl K, Nilsson P, Hirsch JM (2001) Sinus lifting procedure. I. One-stage surgery with bone transplant and implants. Clinical Oral Implants Research 12: 479–487.

46. Kahnberg K-E, Vannas-Löfqvist L (2008) Sinus lift procedure using a 2-stage surgical technique: I. Clinical and radiographic report up to 5 years. Int J Oral Maxillofac Implants 23: 876–884.

47. Kahnberg K-E, Wallström M, Rasmusson L (2011) Local sinus lift for single-tooth implant. I: clinical and radiographic follow-up. Clinical Implant Dentistry and Related Research 13: 231–237.

48. Kaneko T, Masuda I, Horie N, Shimoyama T (2012) New bone formation in nongrafted sinus lifting with space-maintaining management: a novel technique using a titanium bone fixation device. J Oral Maxillofac Surg 70: e217–e224.

49. Keller EE, Eckert SE, Tolman DE (1994) Maxillary antral and nasal one-stage inlay composite bone graft: preliminary report on 30 recipient sites. YJOMS 52: 438–47–discussion447–8.

50. Kermalli JY, Deporter DA, Lai JY, Lam E, Atenafu E (2008) Performance of threaded versus sintered porous-surfaced dental implants using open window or indirect osteotome-mediated sinus elevation: a retrospective report. Journal of Periodontology 79: 728–736.

51. Kim Y-K, Kim S-G, Park J-Y, Yi Y-J, Bae J-H (2011) Comparison of clinical outcomes of sinus bone graft with simultaneous implant placement: 4-month and 6-month final prosthetic loading. Oral Surg Oral Med Oral Pathol Oral Radiol Endod 111: 164–169.

52. Krennmair G, Krainhöfner M, Schmid-Schwap M, Piehslinger E (2007) Maxillary sinus lift for single implant-supported restorations: a clinical study. Int J Oral Maxillofac Implants 22: 351–358.

53. Krennmair G, Krainhöfner M, Piehslinger E (2008) Implant-supported maxillary overdentures retained with milled bars: maxillary anterior versus maxillary posterior concept--a retrospective study. Int J Oral Maxillofac Implants 23: 343–352.

54. Lambert F, Lecloux G, Rompen E (2010) One-step approach for implant placement and subantral bone regeneration using bovine hydroxyapatite: a 2- to 6-year follow-up study. Int J Oral Maxillofac Implants 25: 598–606.

55. Lambrecht JT, Filippi A, Künzel AR, Schiel HJ (2003) Long-term evaluation of submerged and nonsubmerged ITI solid-screw titanium implants: a 10-year life table analysis of 468 implants. Int J Oral Maxillofac Implants 18: 826–834.

56. Leblebicioglu B, Ersanli S, Karabuda C, Tosun T, Gokdeniz H (2005) Radiographic evaluation of dental implants placed using an osteotome technique. Journal of Periodontology 76: 385–390.

57. Lee CYS, Rohrer MD, Prasad HS (2008) Immediate loading of the grafted maxillary sinus using platelet rich plasma and autogenous bone: a preliminary study with histologic and histomorphometric analysis. Implant Dent 17: 59–73.

58. Lee DZ, Chen ST, Darby IB (2012) Maxillary sinus floor elevation and grafting with deproteinized bovine bone mineral: a clinical and histomorphometric study. Clinical Oral Implants Research 23: 918–924.

59. Leick AG, Gulewicz N, Saplacan DI, Broseta ML, Nentwig GH (2005) Einzeitiger Sinuslift mit Knochenersatzmaterial (ESKEM): Klinische Evaluation und metaanalysierender Literaturvergleich. Zeitschrift für Zahnärztliche Implantologie 4: 244.

60. Lin I-C, Gonzalez AM, Chang H-J, Kao S-Y, Chen T-W (2011) A 5-year follow-up of 80 implants in 44 patients placed immediately after the lateral trap-door window procedure to accomplish maxillary sinus elevation without bone grafting. Int J Oral Maxillofac Implants 26: 1079–1086.

61. Lindgren C, Mordenfeld A, Johansson CB, Hallman M (2012) A 3-year clinical follow-up of implants placed in two different biomaterials used for sinus augmentation. Int J Oral Maxillofac Implants 27: 1151–1162.

62. Lundgren S, Andersson S, Gualini F, Sennerby L (2004) Bone reformation with sinus membrane elevation: a new surgical technique for maxillary sinus floor augmentation. Clinical Implant Dentistry and Related Research 6: 165–173.

63. Maiorana C, Redemagni M, Rabagliati M, Salina S (2000) Treatment of maxillary ridge resorption by sinus augmentation with iliac cancellous bone, anorganic bovine bone, and endosseous implants: a clinical and histologic report. Int J Oral Maxillofac Implants 15: 873–878.

64. Maiorana C, Sigurtà D, Mirandola A, Garlini G, Santoro F (2005) Bone resorption around dental implants placed in grafted sinuses: clinical and radiologic follow-up after up to 4 years. Int J Oral Maxillofac Implants 20: 261–266.

65. Mangano C, Bartolucci EG, Mazzocco C (2003) A new porous hydroxyapatite for promotion of bone regeneration in maxillary sinus augmentation: clinical and histologic study in humans. Int J Oral Maxillofac Implants 18: 23–30.

66. Mangano C, Scarano A, Perrotti V, Iezzi G, Piattelli A (2007) Maxillary sinus augmentation with a porous synthetic hydroxyapatite and bovine-derived hydroxyapatite: a comparative clinical and histologic study. Int J Oral Maxillofac Implants 22: 980–986.

67. Marchetti C, Pieri F, Trasarti S, Corinaldesi G, Degidi M (2007) Impact of implant surface and grafting protocol on clinical outcomes of endosseous implants. Int J Oral Maxillofac Implants 22: 399–407.

68. Markovic A, Colić S, Drazić R, Gacić B, Todorović A, et al. (2011) Resonance frequency analysis as a reliable criterion for early loading of sandblasted/acid-etched active surface implants placed by the osteotome sinus floor elevation technique. Int J Oral Maxillofac Implants 26: 718–724.

69. Mazor Z, Peleg M, Garg AK, Chaushu G (2000) The use of hydroxyapatite bone cement for sinus floor augmentation with simultaneous implant placement in the atrophic maxilla. A report of 10 cases. Journal of Periodontology 71: 1187–1194.

70. Mazor Z, Peleg M, Gross M (1999) Sinus augmentation for single-tooth replacement in the posterior maxilla: a 3-year follow-up clinical report. Int J Oral Maxillofac Implants 14: 55–60.

71. Minichetti JC, D'Amore JC, Hong AYJ (2008) Three-year analysis of tapered screw vent implants placed into maxillary sinuses grafted with mineralized bone allograft. J Oral Implantol 34: 135–141.

72. Nedir R, Nurdin N, Vazquez L, Szmukler-Moncler S, Bischof M, et al. (2010) Osteotome sinus floor elevation technique without grafting: a 5-year prospective study. J Clin Periodontol 37: 1023–1028.

73. Peleg M, Garg AK, Mazor Z (2006) Healing in smokers versus nonsmokers: survival rates for sinus floor augmentation with simultaneous implant placement. Int J Oral Maxillofac Implants 21: 551–559.

74. Peleg M, Mazor Z, Chaushu G, Garg AK (1998) Sinus floor augmentation with simultaneous implant placement in the severely atrophic maxilla. Journal of Periodontology 69: 1397–1403.

75. Peleg M, Mazor Z, Garg AK (1999) Augmentation grafting of the maxillary sinus and simultaneous implant placement in patients with 3 to 5 mm of residual alveolar bone height. Int J Oral Maxillofac Implants 14: 549–556.

76. Peleg M, Chaushu G, Mazor Z, Ardekian L, Bakoon M (1999) Radiological findings of the post-sinus lift maxillary sinus: a computerized tomography follow-up. Journal of Periodontology 70: 1564–1573.

77. Pieri F, Aldini NN, Fini M, Marchetti C, Corinaldesi G (2012) Immediate fixed implant rehabilitation of the atrophic edentulous maxilla after bilateral sinus floor augmentation: a 12-month pilot study. Clinical Implant Dentistry and Related Research 14 Suppl 1: e67–e82.

78. Pjetursson BE, Rast C, Brägger U, Schmidlin K, Zwahlen M, et al. (2009) Maxillary sinus floor elevation using the (transalveolar) osteotome technique with or without grafting material. Part I: Implant survival and patients' perception. Clinical Oral Implants Research 20: 667–676.

79. Rodriguez A, Anastassov GE, Lee H, Buchbinder D, Wettan H (2003) Maxillary sinus augmentation with deproteinated bovine bone and platelet rich plasma with simultaneous insertion of endosseous implants. Journal of Oral and Maxillofacial Surgery 61: 157–163.

80. Sakka S, Krenkel C (2011) Simultaneous maxillary sinus lifting and implant placement with autogenous parietal bone graft: outcome of 17 cases. J Craniomaxillofac Surg 39: 187–191.

81. Sbordone L, Levin L, Guidetti F, Sbordone C, Glikman A, et al. (2011) Apical and marginal bone alterations around implants in maxillary sinus augmentation grafted with autogenous bone or bovine bone material and simultaneous or delayed dental implant positioning. Clinical Oral Implants Research 22: 485–491.

82. Scarano A, Piattelli A, Assenza B, Quaranta A, Perrotti V, et al. (2010) Porcine bone used in sinus augmentation procedures: a 5-year retrospective clinical evaluation. J Oral Maxillofac Surg 68: 1869–1873.

83. Schleier P, Bierfreung G, Küpper H, Moldenhauer F, Rabe U, et al. (2006) Die endoskopisch kontrollierte interne Sinusbodenelevation mit simultaner Implantation. Implantologie 1: 65–77.

84. Schleier P, Bierfreund G, Schultze-Mosgau S, Moldenhauer F, Küpper H, et al. (2008) Simultaneous dental implant placement and endoscope-guided internal sinus floor elevation: 2-year post-loading outcomes. Clinical Oral Implants Research 19: 1163–1170.

85. Sforza NM, Marzadori M, Zucchelli G (2008) Simplified osteotome sinus augmentation technique with simultaneous implant placement: a clinical study. Int J Periodontics Restorative Dent 28: 291–299.

86. Siervo S, Ruggli-Milic S, Radici M, Siervo P, Jäger K (2004) [Piezoelectric surgery. An alternative method of minimally invasive surgery]. Schweiz Monatsschr Zahnmed 114: 365–377.

87. Simonpieri A, Choukroun J, Del Corso M, Sammartino G, Dohan Ehrenfest DM (2011) Simultaneous sinus-lift and implantation using microthreaded implants and leukocyte- and platelet-rich fibrin as sole grafting material: a six-year experience. Implant Dent 20: 2–12.

88. Sohn D-S, Heo J-U, Kwak D-H, Kim D-E, Kim J-M, et al. (2011) Bone regeneration in the maxillary sinus using an autologous fibrin-rich block with concentrated growth factors alone. Implant Dent 20: 389–395.

89. Stavropoulos A, Karring T, Kostopoulos L (2007) Fully vs. partially rough implants in maxillary sinus floor augmentation: a randomized-controlled clinical trial. Clinical Oral Implants Research 18: 95–102.

90. Stricker A, Voss PJ, Gutwald R, Schramm A, Schmelzeisen R (2003) Maxillary sinus floor augmention with autogenous bone grafts to enable placement of SLA-surfaced implants: preliminary results after 15-40 months. Clinical Oral Implants Research 14: 207–212.

91. Lee JH, Jung UW, Kim CS, Choi SH, Cho KS (2008) Histologic and clinical evaluation for maxillary sinus augmentation using macroporous biphasic calcium phosphate in human. Clinical Oral Implants Research 19: 767–771.

92. Thor A, Sennerby L, Hirsch JM, Rasmusson L (2007) Bone formation at the maxillary sinus floor following simultaneous elevation of the mucosal lining and implant installation without graft material: an evaluation of 20 patients treated with 44 Astra Tech implants. YJOMS 65: 64–72.

93. Torres J, Tamimi F, Martinez P-P, Alkhraisat MH, Linares R, et al. (2009) Effect of platelet-rich plasma on sinus lifting: a randomized-controlled clinical trial. J Clin Periodontol 36: 677–687.

94. de Vicente JC, Hernández-Vallejo G, Braña-Abascal P, Peña I (2010) Maxillary sinus augmentation with autologous bone harvested from the lateral maxillary wall combined with bovine-derived hydroxyapatite: clinical and histologic observations. Clinical Oral Implants Research 21: 430–438.

95. Viscioni A, Franco M, Paolin A, Cogliati E, Callegari M, et al. (2011) Effectiveness of fresh frozen and cryopreserved homologue iliac crest grafts used in sinus lifting: a comparative study. Cell Tissue Bank 12: 263–271.

96. Voss P, Sauerbier S, Wiedmann-Al-Ahmad M, Zizelmann C, Stricker A, et al. (2010) Bone regeneration in sinus lifts: comparing tissue-engineered bone and iliac bone. Br J Oral Maxillofac Surg 48: 121–126.

97. Wannfors K, Johansson B, Hallman M, Strandkvist T (2000) A prospective randomized study of 1- and 2-stage sinus inlay bone grafts: 1-year follow-up. Int J Oral Maxillofac Implants 15: 625–632.

98. Watzek G, Weber R, Bernhart T, Ulm C, Haas R (1998) Treatment of patients with extreme maxillary atrophy using sinus floor augmentation and implants: preliminary results. International Journal of Oral and Maxillofacial Surgery 27: 428–434.

99. Winter AA, Pollack AS, Odrich RB (2002) Placement of implants in the severely atrophic posterior maxilla using localized management of the sinus floor: a preliminary study. Int J Oral Maxillofac Implants 17: 687–695.

100. Yamada Y, Nakamura S, Ito K, Kohgo T, Hibi H, et al. (2008) Injectable tissue-engineered bone using autogenous bone marrow-derived stromal cells for maxillary sinus augmentation: clinical application report from a 2-6-year follow-up. Tissue Engineering Part A 14: 1699–1707.

101. Yamamichi N, Itose T, Neiva R, Wang H-L (2008) Long-term evaluation of implant survival in augmented sinuses: a case series. Int J Periodontics Restorative Dent 28: 163–169.

102. Hallman M, Zetterqvist L (2004) A 5-year prospective follow-up study of implant-supported fixed prostheses in patients subjected to maxillary sinus floor augmentation with an 80:20 mixture of bovine hydroxyapatite and autogenous bone. Clinical Implant Dentistry and Related Research 6: 82–89.

103. Zijderveld SA, Zerbo IR, van den Bergh JPA, Schulten EAJM, Bruggenkate ten CM (2005) Maxillary sinus floor augmentation using a beta-tricalcium phosphate (Cerasorb) alone compared to autogenous bone grafts. Int J Oral Maxillofac Implants 20: 432–440.

104. Zinner ID, Small SA (1996) Sinus-lift graft: using the maxillary sinuses to support implants. J Am Dent Assoc 127: 51–57.

105. Zitzmann NU, Schärer P (1998) Sinus elevation procedures in the resorbed posterior maxilla. Comparison of the crestal and LATERAL approaches. YMOE 85: 8–17.

106. Zitzmann NU, Schärer P (1998) Sinus elevation procedures in the resorbed posterior maxilla. Comparison of the CRESTAL and lateral approaches. YMOE 85: 8–17.
